# DOING AGING IN PLACE IN AN AGE-FRIENDLY CITY: AN APERTURE TO THE LIVED EXPERIENCES OF OLDER SAN FRANCISCANS

**DOI:** 10.1093/geroni/igz038.099

**Published:** 2019-11-08

**Authors:** Jarmin C Yeh

**Affiliations:** University of California, San Francisco, California, United States

## Abstract

Aging in place finds meaning through the quotidian. The mundanity of this work is the crux of its poignancy. This phenomenological study utilizes photovoice to explore how older adults manage to age in place in an age-friendly city. By interrogating micro- and macro-level realities, this study elicits the strategies seventeen informants use, including how their multiple identities and positionalities become implicated in the process of negotiating and navigating everyday environs, their acts of resistance and resilience, their articulations of hope or pressure to manage the future, as well as the risks and opportunities they encounter and the conditions shaping them, such as urbanization, discrimination, and distribution of resources between generations and groups. To "see" how informants do the “doing” of aging in place has implications for age-friendly community initiatives. It helps to capture the sociality of aging and demonstrates the way the materiality of inequality is sown through lived experience.

